# Inhibitory Effects of Chemical Compounds Isolated from the Rhizome of *Smilax glabra* on Nitric Oxide and Tumor Necrosis Factor-***α*** Production in Lipopolysaccharide-Induced RAW264.7 Cell

**DOI:** 10.1155/2015/602425

**Published:** 2015-03-02

**Authors:** Chuan-li Lu, Wei Zhu, Dong-mei Wang, Wen-long Chen, Meng-mei Hu, Min Wang, Xiao-jie Xu, Chuan-jian Lu

**Affiliations:** ^1^The Second Institute of Clinical Medicine, Guangzhou University of Chinese Medicine, Guangzhou 510120, China; ^2^Guangdong Provincial Academy of Chinese Medical Sciences, Guangzhou 510120, China; ^3^Postdoctoral Research Station, Guangzhou University of Chinese Medicine, Guangzhou 510120, China

## Abstract

The rhizome of *Smilax glabra* has been used for a long time as both food and folk medicine in many countries. The present study focused on the active constituents from the rhizome of *S. glabra*, which possess potential anti-inflammatory activities. As a result, nine known compounds were isolated from the rhizome of *S. glabra* with the bioassay-guiding, and were identified as syringaresinol (**1**), lasiodiplodin (**2**), de-O-methyllasiodiplodin (**3**), syringic acid (**4**), 1,4-bis(4-hydroxy-3,5-dimethoxyphenyl)-2,3-bis(hydroxymethyl)-1,4-butanediol (**5**), lyoniresinol (**6**), *trans*-resveratrol (**7**), *trans*-caffeic acid methyl ester (**8**), and dihydrokaempferol (**9**). Among these compounds, **2** and **3** were isolated for the first time from *S. glabra*. In addition, the potential anti-inflammatory activities of the isolated compounds were evaluated *in vitro* in lipopolysaccharide- (LPS-) induced RAW264.7 cells. Results indicated that **4** and **7** showed significant inhibitory effects on NO production of RAW264.7 cells, and **1**, **2**, **3**, and **5** showed moderate suppression effects on induced NO production. **1**, **7**, and **5** exhibited high inhibitory effects on TNF-*α* production, with the IC_50_ values less than 2.3, 4.4, and 16.6 *μ*M, respectively. These findings strongly suggest that compounds **1**, **2**, **3**, **4**, **5**, **7**, and **9** were the potential anti-inflammatory active compositions of *S. glabra*.

## 1. Introduction

Inflammation is an important physical response to harmful stimuli, such as infection, injury, and irritation [1]. Activated macrophages play key roles in inflammatory diseases related to overproduction of proinflammatory cytokines, including tumor necrosis factor (TNF)-*α*, interleukin- (IL-) 1*β*, IL-6, and inflammatory mediators, including nitric oxide (NO), prostaglandin E_2_ (PGE_2_), and reactive oxygen species (ROS). Thus, inhibiting the production of these macrophage mediators is an important target in treating inflammatory diseases [2]. Bacterial lipopolysaccharide (LPS), the structural component of the Gram-negative bacteria outer cell wall, has been reported to be a major initiator of the inflammatory response during most commonly seen bacterial infections. Binding of LPS to its cognate CD14 receptor on the monocyte/macrophage cell membrane induces the release of various proinflammatory cytokines and chemokines, which are implicated in the pathogenesis of the major inflammatory complications [3].

*Smilax glabra* Roxb, belonging to the family Liliaceae, is a perennial evergreen climbing shrub mainly distributed in China, Japan, Thailand, and so forth [4–6]. This plant is generally consumed as a substitute for tea and sugar to prevent scurvy, and for treating a range of different conditions such as chest ailments, rheumatism, leprosy, impotence, syphilis, and so forth [7]. Syrup made by prolonged boiling of the leaves of* S*.* glabra* was marketed in Sydney in the early 1900s as a tonic and remedy against catarrh and coughs [8]. The rhizome of* S*.* glabra* is named Tufuling in China and commonly consumed in soup, beneficial tea, and herbal medicine. It is also used in folk medicine alone or in combination with other herbal medicines for the treatment of a variety of diseases such as psoriasis and cancer in many other countries.

Previous studies have demonstrated that the rhizome of* S. glabra* possesses a broad spectrum of bioactivities, including hypoglycemic [4], prevention of immunological hepatocyte damage [9], immunomodulatory [10], antiviral [11, 12], antiproliferative [11, 13, 14], and anti-inflammatory activity [13, 15, 16]. Phytochemical investigations on the rhizome of* S*.* glabra* led to the isolation and identification of more than 60 compounds, for example, flavonoids [12, 17–20], phenylpropanoid derivatives [21], and phenolics [12, 22]. In addition, proteins and peptides [23, 24], lectin [25, 26], and glycoproteins [11] were also isolated from the rhizomes of* S. glabra*.

As mentioned above,* S. glabra* has demonstrated a potential to be utilized in health products. However, to the best of our knowledge, phytochemical and pharmacological studies on the edible plant* S. glabra* are limited, and there have been no reports on inhibitory effects of the chemical constituents from* S. glabra* on the proinflammatory mediators. Our previous study indicated that the phenolic-enriched extracts of* S*.* glabra* possessed significant anti-inflammatory activity, in which, astilbin, a known anti-inflammatory compound, was found [27]. The main purpose of the present study was to isolate the chemical constituents of the* S. glabra* rhizomes with bioassay-guiding and evaluate their* in vitro* anti-inflammatory activities in LPS-induced RAW264.7 cells. Overall, aim of the present study was to obtain a comprehensive understanding of the anti-inflammatory compounds in* S. glabra*.

## 2. Materials and Methods

### 2.1. Plant Material

The rhizomes of* S. glabra* were purchased from Kangmei Pharmaceutical Co. Ltd. (Guangdong, China) in February 2013 (batch number 12120527) and were verified by Ph.D. Huang Zhi-hai in the Second Institute of Clinical Medicine, Guangzhou University of Chinese medicine (Guangzhou, China).

### 2.2. Extraction and Isolation

The extraction and isolation of the compounds are shown in Figure 2. In brief, the dried and powdered rhizomes of* S. glabra* (7.0 kg) were extracted with 70% ethanol (90 L × 3) by heating-reflux to give a black crude extract (marked as ESG, 1169.0 g, semidry). ESG (1000 g) was subjected to a HP-20 macroporous resin column by elution with water and 30%, 60%, and 95% ethanol in sequence to give four fractions: ESG-1 (490.0 g), ESG-2 (262.5 g), ESG-3 (116.6 g), and ESG-4 (40.8 g). ESG-2 and ESG-3 showed a significant inhibitory effect on LPS-induced NO production in RAW264.7 cells. ESG-2 and ESG-3 were merged and subjected to column chromatography on silica gel using CH_2_Cl_2_ as the primary eluent with gradual increases in eluent polarity with MeOH to produce 7 subfractions (Frc. 1–7). Further separation of these subfractions using RP-C18 MPLC, preparation HPLC, or/and Sephadex LH-20 chromatography yielded 9 compounds.

### 2.3. Identification of Compounds **1**–**9**

The NMR data of the isolated compounds were recorded on a Bruker AVANCE-500 instrument using TMS as an internal standard. Electrospray ionization mass spectra (ESI-MS) were measured on a Thermo Scientific Finnigan LTQ mass spectrometer, and Preparative HPLC was conducted using a Waters 2545 Binary gradient module instrument with 2998 Photodiode Array Detector and YMC-Pack ODS-A column (250 × 20 mm, 5 *μ*m). Column chromatography (CC) was performed with macroporous adsorption resin Diaion HP-20 (Mitsubishi Chemical Holdings, Japan), silica gel (100–200 mesh, Qingdao Marine Chemical Inc., Qingdao, China), ODS-A (50 *μ*m YMC, Japan), and Sephadex LH-20 (GE Healthcare Bio-Science AB, Sweden). TLC was carried out on glass precoated silica gel GF_254_ plates, and spots were visualized under UV light (254 and/or 366 nm) or by spraying with 10% (v/v) sulfuric acid in ethanol followed by heating to 105°C.

### 2.4. Cell Culture

The mouse macrophage-derived RAW264.7 cell line was purchased from Sun Yat-Sen University, Guangzhou, China, and maintained at 37°C in a humidified atmosphere containing 5% CO_2_ in Dulbecco's modified eagle medium (DMEM) supplemented with 10% heat-inactivated fetal bovine serum (FBS), penicillin (100 U/mL), and streptomycin (100 *μ*g/mL). Cells in exponential growth phase were used for experiments.

### 2.5. Cells Viability Assay

The cytotoxicity of the isolated compounds toward RAW264.7 was evaluated by a conventional MTT assay as reported previously [28]. RAW264.7 cells (1 × 10^5^ cells/well) were inoculated to 96-well plates and incubated for 12 h and then treated with different concentrations of compounds. After additional 24 h incubation, 10 *μ*L of MTT solution (5 *μ*g/mL) was added to each well, and the plate was incubated for another 4 h. The medium was discarded and 150 *μ*L of dimethyl sulfoxide (DMSO) was added to each well, solubilizing formazan. After 15 min incubation, absorbance at 570 nm was read using a microplate reader. The percent viability was calculated using the following formula:

(1) $$\begin{array}{c} \text{Cell}\;\text{viability}\left(\%\right)=\frac{{\text{OD}}_{\text{Control}}-{\text{OD}}_{\text{Sample}}}{{\text{OD}}_{\text{Control}}}\times 100\%. \end{array}$$

### 2.6. Measurement of Nitric Oxide Production in LPS-Induced RAW264.7 Cells

Nitrite accumulation, as an indicator of NO production in the culture medium, was measured with the Griess reagent as reported previously [29]. RAW264.7 cells were plated into 96-well plate at a density of 3 × 10^6^ cells/mL. After 12 h incubation, cells were stimulated by LPS (100 ng/mL) with or without samples for 24 h. Subsequently, the supernatant (100 *μ*L) was harvested and mixed with an equal volume of Griess reagent (0.1%* N*-(1-naphthyl) ethylenediamine dihydrochloride, 1% sulfanilamide, and 2.5% H_3_PO_4_) and stood for 15 min at room temperature in the dark. NO production was determined by measuring absorbance at 540 nm and was converted to nitrite concentration by reference to a standard curve generated with sodium nitrite.

### 2.7. Determination of TNF-*α* Production in LPS-Induced RAW264.7 Cells

RAW264.7 cells (3 × 10^6^ cells/mL) were seeded onto 24-well culture plate and incubated for 12 h. The cells were then pretreated with various concentrations of the isolated compounds for 2 h before stimulation with LPS (100 ng/mL) with or without samples for 12 h. Supernatants were then collected and the TNF-*α* concentrations in the medium were determined using commercially available ESISA kits according to the manufacturer's instructions as described in previous study [29].

### 2.8. Statistical Analysis

All values in the figures and text were expressed as means ± SD. The results were analyzed by one-way ANOVA. All analyses were performed using the Statistical Package for the Social Sciences (SPSS) software. A *P* value less than 0.05 was considered significant.

## 3. Results and Discussion

**Figure 1:**
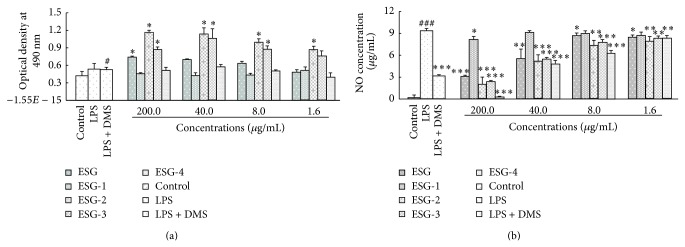
Effects of ethanol extract of* S. glabra* (ESG) and its four subfractions (ESG-1, ESG-2, ESG-3, and ESG-4) on RAW264.7 cells viability and LPS-induced NO production. Cells viability (a) was tested by MTT assay; higher value of optical density at 490 nm means higher cell viability. Nitrite accumulated in cell culture supernatants was determined by Griess assay as an index for NO release (b). Cells were treated with LPS (1 *μ*g/mL) for 24 h in the absence or presence of samples (1.6, 8.0, 40, and 200.0 *μ*g/mL). The data were presented as mean ± S.D. (*n* = 5). Dexamethasone (DMS) was employed as a positive control. # and ###, respectively, mean *P* < 0.05 and *P* < 0.001 compared with control group. ∗, ∗∗, and ∗∗∗, respectively, mean *P* < 0.05, *P* < 0.01, and *P* < 0.001 compared with group treated with LPS alone.

**Figure 2:**
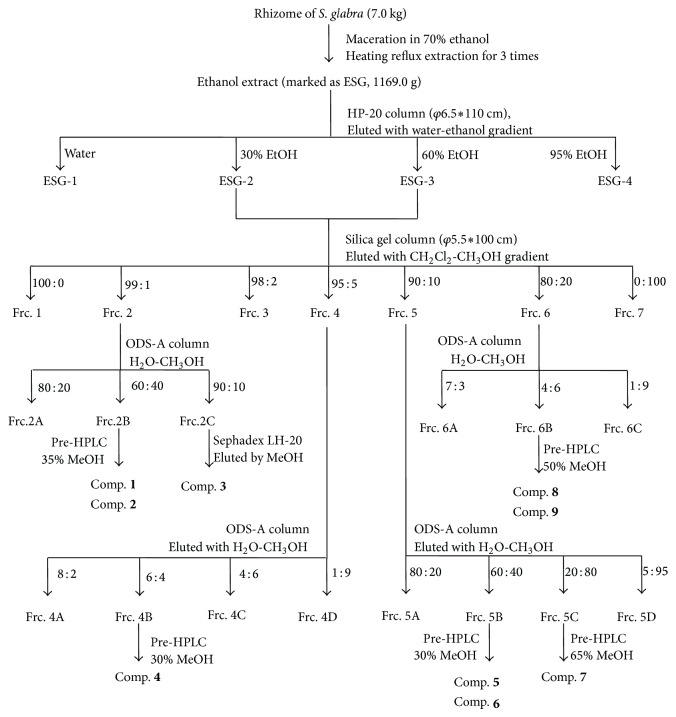
Purification processes of compounds** 1**–**9**.

The dried rhizomes of* S*.* glabra* were extracted with 70% ethanol by heating-reflux to give a black crude extract (marketed as ESG), which was further partitioned into four fractions (ESG-1, ESG-2, ESG-3, and ESG-4) by subjecting to a HP-20 macroporous resin column with gradient elution of ethanol-water. Since fractions ESG-2 and ESG-3 showed a significant inhibitory effect on LPS-induced NO production in RAW264.7 cells (Figure 1), they were merged and further subjected to various column chromatographies to yield 9 known compounds (Figure 2).

**Figure 3:**
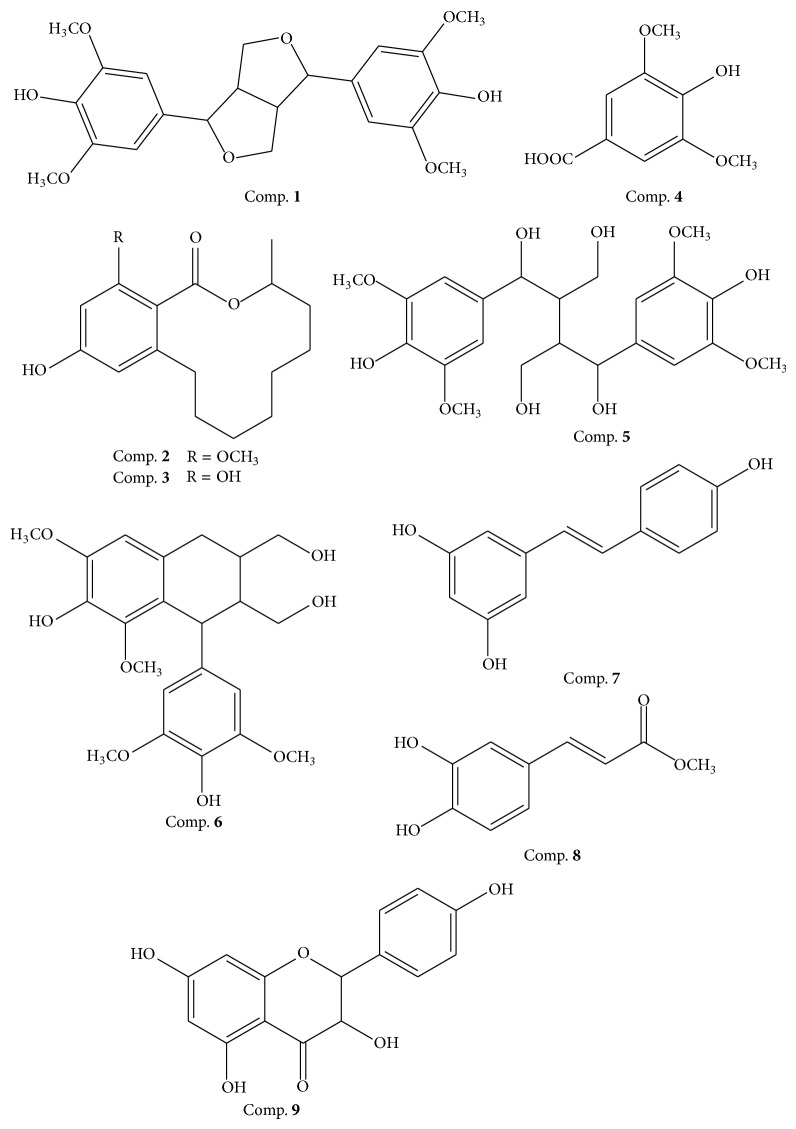
Structures of compounds** 1**–**9**.

The structures of these compounds were identified, based on the establishing ESI-MS and NMR data, as syringaresinol (**1**), lasiodiplodin (**2**), de-O-methyllasiodiplodin (**3**), syringic acid (**4**), 1,4-bis(4-hydroxy-3,5-dimethoxyphenyl)-2,3-bis(hydroxymethyl)-1,4-butanediol (**5**), lyoniresinol (**6**),* trans*-resveratrol (**7**),* trans*-caffeic acid methyl ester (**8**), and dihydrokaempferol (**9**), respectively (Figure 3). Among these compounds,** 2** and** 3** were for the first time isolated from the rhizome of* S*.* glabra*. The spectral data of the isolated compounds are described below.

*Syringaresinol* ***(1)*** *, Colourless Amorphous Powder*. Its positive-ion ESI-MS (*m/z*) displayed quasi-molecular ion peaks at 419 [M + H]^+^, 441 [M + Na]^+^, and 859 [2M + Na]^+^, indicating a molecular weight of 418. ^1^H-NMR (500 MHz, CD_3_OD) *δ*_H_ 6.62 (4H, s, H-2′, 2′′, 6′ and 6′′), 4.70 (2H,* brd*, *J* = 4.3 Hz, H-2, H-6), 4.24 (2H,* dd J* = 6.8, 9.0 Hz, H-4, H-8), 3.86 (2H,* dd J* = 3.8, 9.0 Hz, H-4, H-8), 3.83 (12H,* s*, 4 × OCH_3_), and 3.12 (2H,* m*, H-1 and 5). ^13^C-NMR (125 MHz, CD_3_OD) *δ*_C_ 149.3 (C-3′, 3′′, 5′, and 5′′), 136.2 (C-1′ and 1′′), 133.1 (C-4′ and 4′′), 104.4 (C-2′, 2′′, 6′, and 6′′), 87.6 (C-2 and 6), 72.7 (C-4 and 8), 56.8 (4 × OCH_3_), and 55.5 (C-1 and 5). Based on these ^1^H NMR and ^13^C NMR data, compound** 1** was identified as syringaresinol by comparison with the data reported previously [30].

*Lasiodiplodin* ***(2)*** *, White Needles (MeOH)*. Its positive-ion ESI-MS (*m/z*) displayed quasi-molecular ion peaks at 339 [M + H]^+^, 361 [M + Na]^+^, and negative-ion ESI-MS (*m/z*) 337 [M − H]^−^, indicating a molecular weight of 338. ^1^H-NMR (500 MHz, CD_3_OD) *δ*_H_ 1.25–2.18 (12H,* m*, H4–9), 1.32 (3H,* d*, *J* = 6.5 Hz, H-17), 2.48–2.70 (2H,* m*, H-10), 3.77 (3H, s, –OCH_3_), 5.18 (1H,* m*, H-3), 6.30 (1H, d, *J* = 2.0 Hz, H-12), 6.26 (1H, d, *J* = 2.0 Hz, H-14). ^13^C-NMR (125 MHz, CD_3_OD) *δ*_C_ 171.1 (C-1), 160.9 (C-13), 159.6 (C-15), 144.0 (C-11), 117.8 (C-16), 109.3 (C-12), 98.0 (C-14), 73.6 (C-3), 56.4 (C-18), 33.8 (C-4), 31.4 (C-10), 31.2 (C-9), 27.8 (C-6), 26.5 (C-8), 25.7 (C-7), 22.5 (C-5), and 20.1 (C-17). The data of ^1^H NMR and ^13^C NMR were consistent with those in [31]. Compound** 2** was identified as lasiodiplodin.

*de-O-Methyllasiodiplodin* ***(3)*** *, White Needles (MeOH).* Its positive-ion ESI-MS (*m/z*) displayed quasi-molecular ion peaks at 325 [M + H]^+^, 347 [M + Na]^+^, and negative-ion ESI-MS (*m/z*) 323 [M − H]^−^, indicating a molecular weight of 324. ^1^H-NMR (500 MHz, CD_3_OD) *δ*_H_ 1.39–2.00 (12H,* m*, H4–9), 1.36 (3H,* d*, *J* = 6.5 Hz, H-17), 2.42–2.70 (2H,* m*, H-10), 5.17 (1H,* m*, H-3), 6.21 (1H, d, *J* = 2.0 Hz, H-12), 6.16 (1H, d, *J* = 2.0 Hz, H-14). ^13^C-NMR (125 MHz, CD_3_OD) *δ*_C_ 171.2 (C-1), 164.0 (C-13), 156.6 (C-15), 147.8 (C-11), 110.0 (C-16), 104.2 (C-12), 99.9 (C-14), 74.0 (C-3), 32.3 (C-4), 30.3 (C-10), 30.1 (C-9), 26.3 (C-6), 23.8 (C-8), 23.6 (C-7), 20.6 (C-5), and 18.6 (C-17). The ^1^H NMR and ^13^C NMR data of compound** 2** was similar with that of compound** 2**, but showed no methoxyl-signals. The molecular weight of compound** 3** was 14 less than that of compound** 2**. Based on these NMR and ESI-MS data, compound** 3** was identified as de-O-methyllasiodiplodin by comparison with the data reported previously [31].

*Syringic Acid* ***(4)*** *, White Needles (MeOH)*. Its positive-ion ESI-MS (*m/z*) displayed quasi-molecular ion peaks at 199 [M + H]^+^, 221 [M + Na]^+^, 237 [M + K]^+^, and negative-ion ESI-MS (*m/z*) 197 [M − H]^−^, 233 [M + Cl]^−^ indicating a molecular weight of 198. ^1^H-NMR (500 MHz, CD_3_OD) *δ*_H_ 7.33 (2H, s, H-2 and 6), 3.88 (3H, s, 2 × –OCH_3_). ^13^C-NMR (125 MHz, CD_3_OD) *δ*_C_ 170.5 (–COOH), 149.0 (C-3 and 5), 141.7 (C-4), 122.4 (C-1), 108.0 (C-2 and 6), 56.9 (2 × –OCH_3_). All spectral data agreed with that previously reported [32].

*1,4-Bis(4-hydroxy-3,5-dimethoxyphenyl)-2,3-bis(hydroxymethyl)-1,4-butanediol* ***(5)*** *, White Amorphous Powder*. ESI-MS:* m/z* 481 [M − H]^−^ and 483 [M + H]^+^ indicated a molecular weight of 482. ^1^H-NMR (500 MHz, CD_3_OD) *δ*_H_ 6.75 (4H, s, H-2, 2′, 6, 6′), 4.97 (2H, d, *J* = 8.5 Hz, H-7, 7′), 3.87 (12H, s, –OCH_3_  × 4), 3.70–3.62 (4H, m, H-9, 9′), 2.35 (2H, m, H-8, 8′). ^13^C-NMR (125 MHz, CD_3_OD) *δ*_C_ 149.4 (C-3, 5, 3′, 5′), 136.0 (C-4, 4′), 134.5 (C-1, 1′), 104.9 (C-2, 6, 2′, 6′), 84.7 (C-7, 7′), 61.7 (C-9, 9′), 54.9 (C-8, 8′). The data of ^1^H NMR and ^13^C NMR were consistent with those in [12].

*Lyoniresinol* ***(6)*** *, White Amorphous Powder*. ESI-MS:* m/z* 421 [M + H]^+^, 443 [M + Na]^+^, and 841 [2M + H]^+^ indicated a molecular weight of 420. ^1^H-NMR (500 MHz, CD_3_OD) *δ*_H_ 6.58 (1H,* s*, H-8), 6.39 (2H,* s*, H-2′ and 6′), 4.31 (1H,* d*, *J* = 5.7 Hz, H-4), 3.85 (3H, s, 3-OCH_3_), 3.74 (6H,* s*, 5,7-OCH_3_), 3.59 (1H, m, H-2*α*), 3.48 (1H, m, H-3*α*), 2.60 (2H, m, H-1), 1.95 (1H, m, H-3). ^13^C-NMR (125 MHz, CD_3_OD) *δ*_C_ 149.1 (C-3′, 5′), 148.8 (C-5), 147.8 (C-7), 139.5 (C-1′), 139.0 (C-6), 134.6 (C-4′), 130.3 (C-9), 126.4 (C-10), 107.9 (C-8), 106.9 (C-2′, 6′), 66.9 (C-3*α*), 64.2 (C-2*α*), 60.3 (5-OCH_3_), 56.9 (C-3′, 5′-OCH_3_), 56.7 (7-OCH_3_), 42.5 (C-3), 41.0 (C-2), and 33.7 (C-1). The data of ^1^H NMR and ^13^C NMR were consistent with those in [33].

*trans-Resveratrol* ***(7)*** *, White Needles (MeOH)*. ESI-MS:* m/z* 227 [M − H]^−^, 455 [2M − H]^−^, and 229 [M + H]^+^ indicated a molecular weight of 228. ^1^H-NMR (500 MHz, CD_3_OD) *δ*_H_ 7.35 (2H,* d*, *J* = 8.5 Hz, H-2′, 6′), 6.94 (1H,* d*, *J* = 16.5 Hz, H-1), 6.80 (1H,* d*, *J* = 16.5 Hz, H-2), 6.77 (2H,* d*, *J* = 8.5 Hz, H-3′, 5′), 6.46 (2H,* d*, *J* = 2.5 Hz, H-2′′, 6′′), 6.17 (1H,* t*, *J* = 2.5 Hz, H-4′′). ^13^C-NMR (125 MHz, CD_3_OD) *δ*_C_ 159.8 (C-3′′, 5′′), 158.5 (C-4′), 141.5 (C-1′′), 130.6 (C-1′), 129.6 (C-1), 129.0 (C-2′, 6′), 127.1 (C-2), 116.0 (C-3′, 5′), 105.9 (C-2′′, 6′′), 102.8 (C-4′′). Based on these ^1^H NMR and ^13^C NMR data, compound** 9** was identified as* trans*-resveratrol by comparison with the data reported previously [34].

*trans-Caffeic Acid Methyl Ester* ***(8)*** *, White Amorphous Powder.* ESI-MS:* m/z* 193 [M − H]^−^, 229 [M + Cl]^−^, 387 [2M − H]^−^, and 195 [M + H]^+^ indicated a molecular weight of 194. ^1^H-NMR (500 MHz, CD_3_OD) *δ*_H_ 7.52 (1H,* d*, *J* = 15.9 Hz, H-7), 7.02 (1H,* d*, *J* = 1.7 Hz, H-2), 6.92 (1H,* dd*, *J* = 1.7, 8.2 Hz, H-5), 6.76 (1H,* d*, *J* = 8.2 Hz, H-6), 6.42 (1H,* d*, *J* = 15.9 Hz, H-8), 3.73 (3H,* s*, –OCH_3_). ^13^C-NMR (125 MHz, CD_3_OD) *δ*_C_ 169.7 (C-9), 149.5 (C-7), 146.9 (C-4), 146.8 (C-3), 127.6 (C-1), 122.9 (C-6), 116.4 (C-5), 115.1 (C-2), 114.8 (C-8), 52.0 (–OCH_3_). The data of ^1^H NMR and ^13^C NMR were consistent with those in [35].

*Dihydrokaempferol* ***(9)*** *, Colourless Amorphous Powder.* ESI-MS:* m/z* 287 [M − H]^−^, 323 [M + Cl]^−^, and 289 [M + H]^+^ indicated a molecular weight of 288. ^1^H-NMR (500 MHz, CD_3_OD) *δ*_H_ 7.35 (2H,* d*, *J* = 8.5 Hz, H-2′, 6′), 6.83 (2H,* d*, *J* = 8.5 Hz, H-3′, 5′), 5.83 (2H, br*s*, H-6, 8), 4.95 (1H,* d*, *J* = 11.5 Hz, H-2), 4.50 (1H,* d*, *J* = 11.5 Hz, H-3). ^13^C-NMR (125 MHz, CD_3_OD) *δ*_C_ 197.8 (C-4), 164.9 (C-7), 164.6 (C-5, 9), 159.3 (C-4′), 130.5 (C-2′, 6′), 129.6 (C-1′), 116.2 (C-3′, 5′), 101.6 (C-10), 97.2 (C-6, 8), 85.0 (C-2), 73.7 (C-3). The data of ^1^H NMR and ^13^C NMR were consistent with those in [36].

NO is well known as endogenous regulators of cell and tissue function, but excessive production of NO maybe participate in several autoimmune or chronic inflammatory diseases [37]. TNF-*α* is the earliest and primary endogenous mediator of the process of inflammatory reaction and mediates the inflammatory response the local and systemic levels [38]. Therefore, the inhibition of these media can be a very important target for development of anti-inflammation agents.

The anti-inflammatory activities of the isolates were tested* in vitro* for inhibiting the NO and TNF-*α* production in LPS-induced RAW264.7 cells. Firstly, the cytotoxicity of these compounds cells on the proliferation of RAW264.7 cells was measured by MTT assay. The IC_10_ value of each compound (Table 1), at which 10% of cells proliferation was inhibited, was calculated with an improved Karber methodology and defined as the highest noncytotoxic concentration. While the dose is ≤IC_10_, the effect of the isolated compound on RAW264.7 cells could be neglected. In the following experiments, the concentrations of each compound were set at [IC_10_] × 1, [IC_10_] × 10^−1^, and [IC_10_] × 10^−2^ levels.

**Figure 4:**
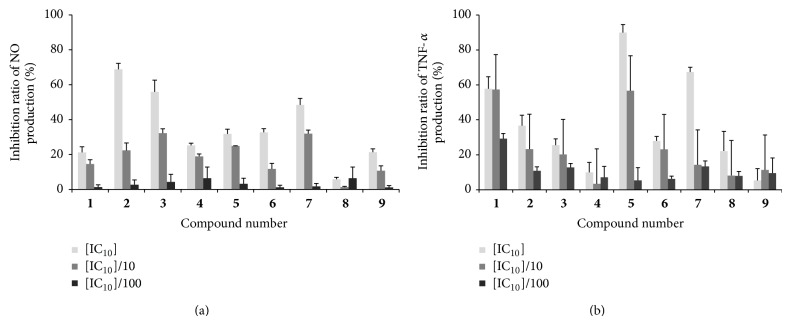
The inhibitory effects of the isolated compounds on NO (a) and TNF-*α* (b) productions in LPS-induced RAW264.7 cells. Cells were seeded into 24-well plates and incubated for 12 h and then treated with or without different concentrations of compounds for 24 h (for NO) or 14 h (for TNF-*α*). The culture supernatant was analyzed for nitrite and TNF-*α* production. Results shown are representative of three separate experiments. All conditions were run in triplicate, and data shows mean ± SD values.

As shown in Figure 4(a), all isolated compounds, expect Comp.** 8**, exhibited dose-dependent inhibitory effects on LPS-induced NO production in RAW264.7 cells. Particularly, Comp.** 7** at 4.4 and 0.4 *μ*M, respectively, inhibited about 48% and 32% of LPS-induced NO production, while Comp.** 4** at 5.1 and 0.5 *μ*M inhibited about 25% and 19% of induced NO production, respectively. Compounds** 1**,** 2**,** 3**, and** 5** showed moderate suppression effects on induced NO production. But, Compounds** 6** and** 9** showed the lowest inhibitory effects, at level of IC_10_, respectively, 238.1 and 139.4 *μ*M; just 32.7% and 21.3% of NO production were inhibited separately. The inhibitory effects of isolated compounds on TNF-*α* production presented in Figure 4(b) showed that all isolated compounds, expect Compounds** 4** and** 9**, exhibited dose-dependent inhibitory effects on LPS-induced TNF-*α* production in RAW264.7 cells. Compounds** 1**,** 7**, and** 5** exhibited high inhibitory effects on TNF-*α* production, with the IC_50_ values less than 2.3, 4.4, and 16.6 *μ*M, respectively.

The results of the present study were consistent with that reported previously on the anti-inflammatory activities of the isolated compounds. Resveratrol is a natural polyphenolic stilbene derivative found in a variety of edible fruits and is known for its multiple pharmacological activities. Its anti-inflammatory activity has been demonstrated* in vitro* and* in vivo*, by showing that it could attenuate cytokine production in adipose tissue by repressing TLR2- and TLR4-mediated proinflammatory signaling cascades and decrease COX-2 expression [39]. Syringaresinol isolated from many different plants has been demonstrated to possess significant anti-inflammatory activity, which could significantly inhibit NO, PGE2, and TNF-*α* production of LPS-induced RAW264.7 and BV-2 cells, as well as decrease the expression level of iNOS and COX-2 enzyme [40–42]. And other compounds, including syringic acid and dihydrokaempferol were also reported previously to exhibit potential anti-inflammatory activities [36, 43, 44].

Taken together, the present study demonstrated that* trans*-resveratrol, syringic acid, syringaresinol, lasiodiplodin, de-O-methyllasiodiplodin, and dihydrokaempferol are the potential anti-inflammatory active constituents of the rhizomes of* S. glabra*. Further studies should be carried out to evaluate their anti-inflammatory effects* in vivo* and the mechanisms of action by which effects are mediated.

## Figures and Tables

**Table 1:** The inhibitory effects of the isolated compounds on RAW264.7 cells.

Comp.	IC_10_ values (*μ*mol/L)
Comp. **1**	23.9 ± 1.8
Comp. **2**	147.9 ± 15.4
Comp. **3**	154.3 ± 20.5
Comp. **4**	5.1 ± 4.2
Comp. **5**	166.0 ± 9.7
Comp. **6**	238.1 ± 31.6
Comp. **7**	4.4 ± 5.7
Comp. **8**	5.2 ± 3.2
Comp. **9**	139.4 ± 10.3

Cells were seeded into 96-well plates and incubated for 12 h and then treated with or without different concentrations of compounds for 24 h incubation. After that, MTT assay was employed to determine the cells viability. All experiments were run in triplicate, and IC_10_ values were calculated with an improved Karber methodology.
